# Supplementary material for: The interactome of intact mitochondria by cross-linking mass spectrometry provides evidence for coexisting respiratory supercomplexes
Source: Mol Cell Proteomics. 2017 Dec 8;17(2):216–32. doi: 10.1074/mcp.RA117.000470 (PMC5795388; doi:10.1074/mcp.RA117.000470)
Supplement: Supplemental Data [file supp_RA117.000470_133922_0_supp_23978_fzffwf.zip › spectra_annotation/mito_DR_spectra_annotation/20-1-4-1-19-1.pdf]

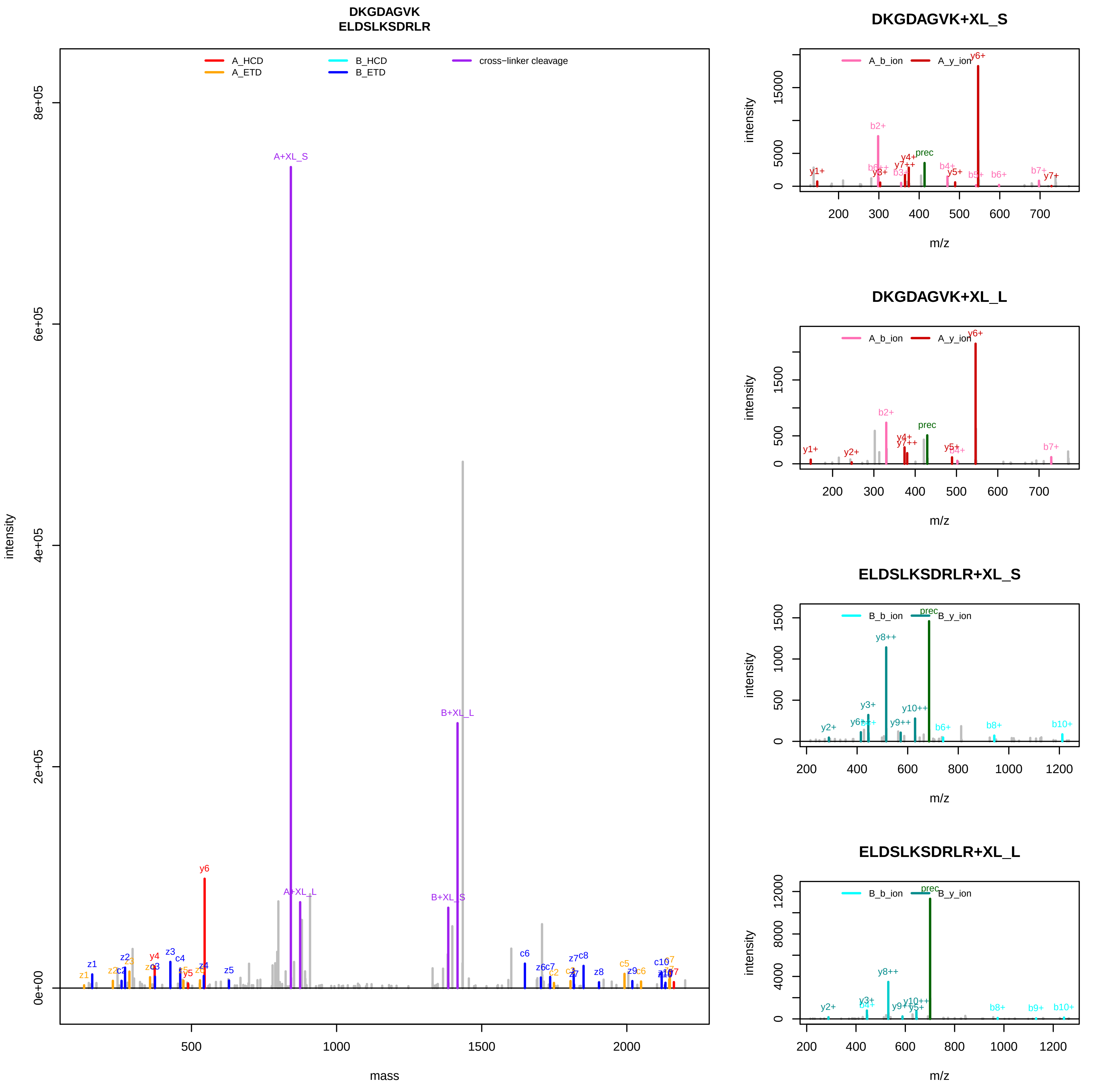

DKGDAGVK
ELDSLKSDRLR
A_HCD
A_ETD
B_HCD
B_ETD
cross−linker cleavage
intensity
mass
0e+00
2e+05
4e+05
6e+05
8e+05
500
1000
1500
2000
A+XL_S
A+XL_L
B+XL_L
B+XL_S
y6
y4
y5
z1
z2
c2
z3
c3
c4
z4
z5
c6
z6
c7
z7
c8
z8
z9
c10
z10
c5
DKGDAGVK+XL_S
DKGDAGVK+XL_L
A_b_ion
A_y_ion
y6+
b2+
prec
y1+
y2+
y3+
y4+
y5+
y7+
y7++
b3+
b4+
b5+
b6+
b6++
b7+
ELDSLKSDRLR+XL_S
ELDSLKSDRLR+XL_L
B_b_ion
B_y_ion
y8++
y3+
y2+
y6+
y9++
y9+
y10++
y5+
b4+
b6+
b8+
b9+
b10+
m/z
intensity
0
500
1000
1500
5000
15000
4000
8000
12000
200
300
400
500
600
700
800
1200
